# Sciatic Schwannoma in a Patient with Neurofibromatosis Type 1: A Report of a Minimally Invasive Surgical Technique and Review of the Literature

**DOI:** 10.7759/cureus.101058

**Published:** 2026-01-07

**Authors:** Eric O Sarpong, Ramin Rahmanzade, Adolf Mueller, Hannes Egermann, Attila Sarkadi

**Affiliations:** 1 Neurosurgery, Barmherzige Brüder Hospital Regensburg, Regensburg, DEU; 2 Pathology, LMU University Hospital, Munich, DEU; 3 Neurosurgery, Klinikum Barmherzige Brüder Regensburg, Regensburg , DEU

**Keywords:** minimal invasive approach, neurofibromatosis type 1 (nf-1), neurofibromatosis type two, peripheral nerve sheath tumor, sciatica pain, sciatic schwannoma

## Abstract

Schwannomas rarely occur in patients with neurofibromatosis type 1 (NF1). A sciatic schwannoma (SS) is the cause of sciatica in less than one percent of cases. These tumors are traditionally resected by the transgluteal approach, gaining access to the tumor through a large incision. Herein, we report a minimally invasive resection of an SS in a patient with NF1, who presented with typical sciatica four months after resection of a lumbar schwannoma. The clinical and imaging characteristics of these tumors are also reviewed.

## Introduction

Schwannomas and neurofibromas are peripheral nerve sheath tumors (PNSTs) that occur on the peripheral nerves as solitary tumors or in association with neurocutaneous syndromes, namely, neurofibromatosis (NF) [1]. NF is commonly associated with schwannomas in NF2 and neurofibromas in NF1 of the peripheral nerves [1]. Patients with NF1 typically present with two or more clinical features, including café-au-lait macules, freckling predominantly in the axillary and inguinal regions, Lisch nodules, peripheral neurofibromas, and optic gliomas, among others [2]. Patients with NF1 presenting with schwannomas of the peripheral nerves and spine are rare [3]. In contrast, NF2 is characterized by multiple inherited schwannomas, meningiomas, and ependymomas [2]. In addition, schwannomas usually occur sporadically, whereas neurofibromas typically occur as part of neurofibromatosis, most often associated with NF1 and only occasionally with NF2 or schwannomatosis [1].

The tumor burden in patients with NF is higher in the pelvis and leg regions compared to other body regions [1]. In analyzing the management of 175 patients with 201 peripheral nerve tumors, Guha et al. showed that tumor distribution is similarly higher in the pelvis and lower extremities [1]. For tumor distribution in the lower extremity, sciatic nerve tumors were the most dominant [1].

These tumors may clinically cause sciatica, which has been considered one of the most common medical complaints worldwide [4]. It typically manifests with lumbosacral pain radiating unilaterally to the dorsolateral aspect of the lower extremity [4]. Although sciatica mainly occurs due to the compression of spinal nerve roots or trunks, other less common causes, such as sciatic neurinoma, should be taken into account, especially in patients with atypical presentations or disease courses [5]. Sciatic tumors are the cause of the sciatica of nondisc origin in only 1.7% of cases in a large series of 239 patients [6]. Sciatic schwannoma (SS) accounts for less than 1% of cases of sciatica [6]. Although the radicular pain associated with SS originates not from the lumbosacral region but from the thigh, it radiates ipsilaterally along the distal distribution of the sciatic nerve, similar to sciatica commonly caused by lumbar disc herniation [4]. An extremely low rate of recurrence and a postoperative course independent of patients’ lifestyle and physical activities, unlike sciatica caused by disk herniation, suggest the key role of surgical resection in the lifelong resolution of symptoms when SS is the cause [1,7].

We report the case of a patient with NF1 and multiple schwannomas. Due to the location and imaging characteristics of SS, we herein introduce our minimally invasive tubular approach for its resection and review this relatively unknown entity, highlighting when suspicion for these tumors should be raised and discussing diagnostic and therapeutic approaches. The patient’s medical records were thoroughly reviewed and analyzed in the context of current literature.

## Case presentation

Clinical presentation

A 40-year-old man with a known history of NF type 1 (NF1) presented to our outpatient clinic with severe back pain radiating intermittently to the posterolateral aspect of the right thigh for the past two weeks. He also complained of dysesthesia in the same region. The patient's medical history included multiple café au lait macules on the trunk, neurofibromas in the nipple region, and mental retardation. There were no associated sensory or motor deficits, bladder dysfunction, or history of trauma. He additionally reported a past diagnosis of carpal tunnel syndrome. On clinical examination, he exhibited a limping gait and a positive Lasègue’s sign, suggestive of sciatic nerve irritation. Cutaneous stigmata of NF1, such as café au lait macules and neurofibromas, were evident on inspection.

**Figure 1:**
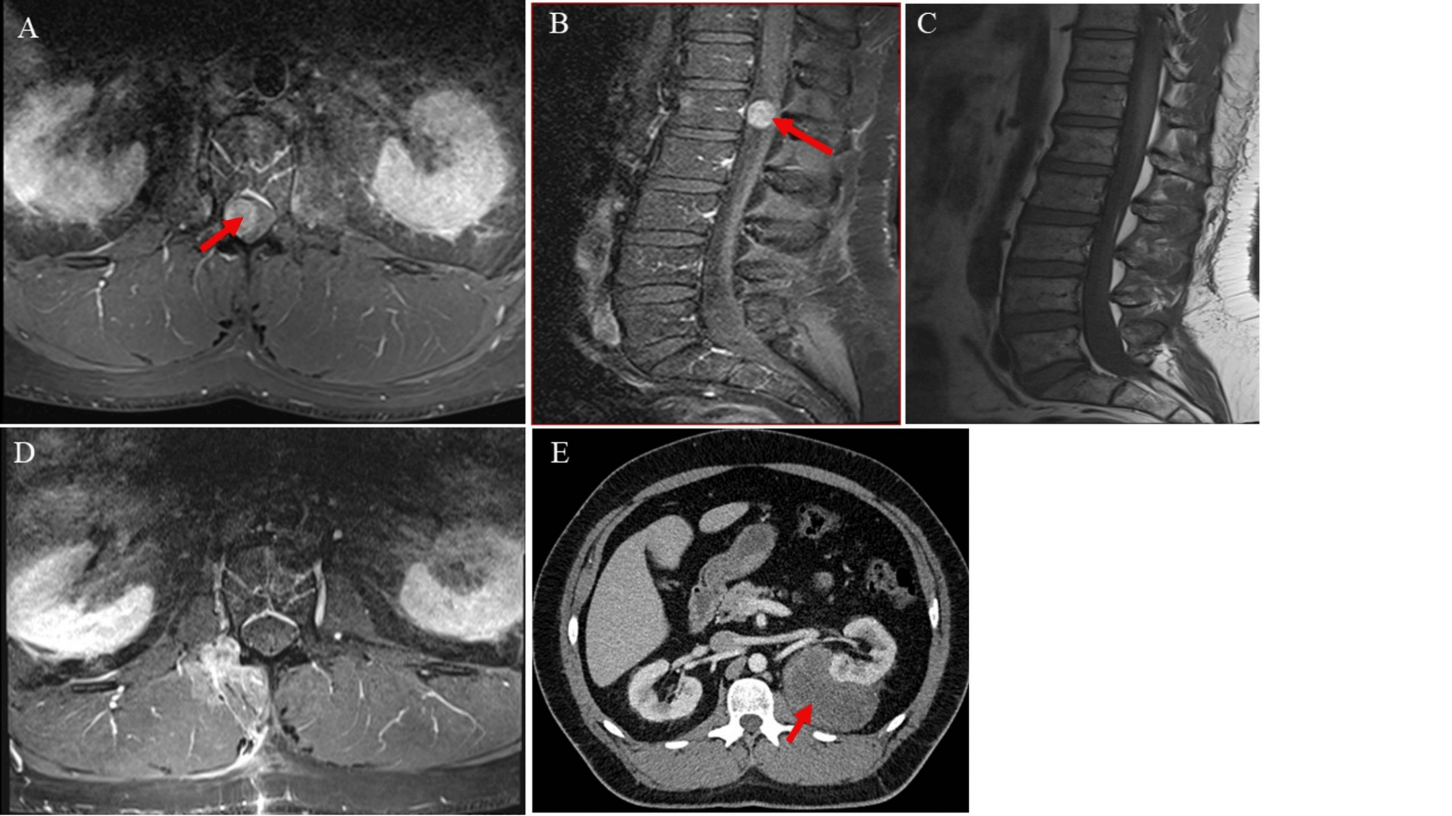
Images of the mass lesions: lumbar region (pre- and postoperative) and perirenal region. (A, B) Axial and sagittal T1-weighted MRI of the lumbar spine with contrast demonstrating a well-defined, homogeneously contrast-enhancing spinal mass at the L1/L2 level, measuring 3.5 cm transversely, 1.5 cm anteroposteriorly, and 2.5 cm cranio-caudally (red arrow). (C, D) Axial and sagittal postoperative T1-weighted MRI showing no recurrent mass at L1/L2, consistent with gross total resection of the schwannoma (red). (E) Contrast-enhanced CT of the abdomen demonstrating a large, heterogeneous retroperitoneal mass measuring 96 × 93 × 47 mm (red arrow), consistent with a perirenal liposarcoma.

Magnetic resonance imaging (MRI) of the lumbar spine revealed a well-circumscribed spinal lesion at the level of L1/L2 that demonstrated hypointense signal on T1-weighted sequences and marked enhancement following gadolinium administration, consistent with a peripheral nerve sheath tumor such as a schwannoma (Figures 1A-1B).

The tumor was resected via a dorsolateral transmuscular approach at the L1/L2 level, using intraoperative neuromonitoring to preserve surrounding nerve function. The histopathological examination of the excised specimen demonstrated the classic biphasic architecture consistent with schwannoma, with alternating Antoni A and Antoni B regions. The patient´s preoperative symptoms resolved following surgery, and his postoperative course was unremarkable. Postoperative MRI study confirmed the gross total resection of the tumor (Figures 1C-1D).

Four months later, the patient complained of a recurrence of similar symptoms, reporting intermittent pain and dysesthesia in the posterolateral aspect of the right thigh, raising concern for tumor recurrence.

MRI of the lumbar spine revealed a contrast-enhancing lesion at the L1/L2 level following gadolinium administration (Figures 1A-1B).

**Figure 2:**
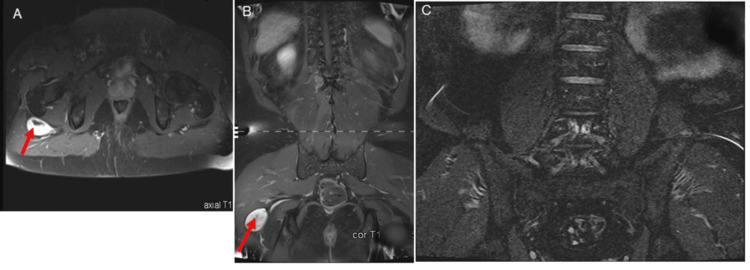
Preoperative and postoperative MRI images of the sciatic schwannoma. (A, B) Coronal and axial T1-weighted MRI of the lumbar spine and proximal thigh region with contrast, demonstrating a well-defined mass along the course of the right sciatic nerve, located posterior to the greater trochanter and ventral to the gluteus maximus muscle. The lesion measures approximately 4.7 × 2.4 × 5 cm (red arrow). (C) Postoperative MRI using an STIR sequence of the same region demonstrating no residual or recurrent mass, with expected postoperative changes in the soft tissues and surrounding musculature after gross total resection of the schwannoma.

**Figure 3:**
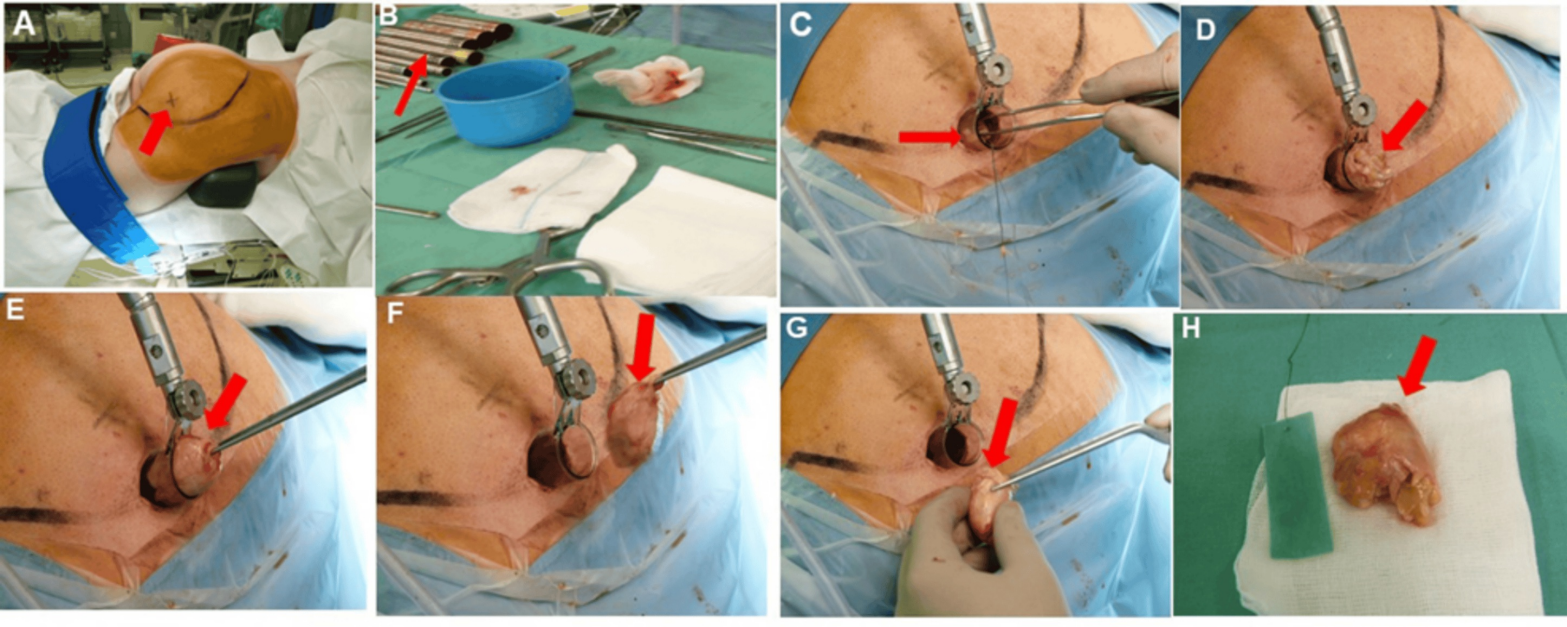
Surgical images depicting the minimally invasive technique used to resect the sciatic schwannoma. (A) Image showing the disinfected and exposed right gluteal region. The tumor area is marked along the gluteal muscle (arrow). (B) The arrow points to the series of tubular dilators used. (C) The right gluteal region is draped, and the tubular retractor system is in place. The tumor has been carefully dissected under intraoperative neuromonitoring and is being removed with forceps. (E, F, G) Views of the tumor within the tubular retractor being removed with forceps (arrows). (H) The completely removed tumor, approximately 5 cm in diameter.

There was no complaint of back pain, no sensory or motor deficit, and no bladder dysfunction. The MRI study of the lumbar spine excluded postoperative complications. MRI of the thigh region revealed a 4 cm x 4 cm mass located posterior to the greater trochanter and ventral to the gluteus maximus muscle (Figures 2A-2B), consistent with a peripheral nerve tumor; the patient was subsequently admitted for surgery. A tubular, minimally invasive transgluteal approach was used to resect the tumor with minimal collateral injuries (Figure 3) under neuromonitoring. The postoperative pain was managed with analgesics. Histological examination of the excised tumor confirmed the diagnosis of an SS. At the last follow-up, seven years after surgery, the patient remained asymptomatic, with complete resolution of pain and no evidence of tumor recurrence on MRI (Figure 2C). The patient was later also diagnosed with a perirenal liposarcoma (Figure 1E), which was subsequently completely resected.

Minimally invasive surgical technique

The patient was placed in the supine position under general anesthesia (Figure 3A). To localize the tumor, an ultrasonographic examination of the dorsolateral aspect of the right thigh was carried out. It revealed a centrally hypoechoic mass with an echogenic periphery located eccentrically on the right sciatic nerve, highly suggestive of a schwannoma. After prepping and draping, a 2.5 cm vertical incision was made, equal in length to the diameter of the retractor (Figure 3B). The cannulated soft tissue dilator was placed through the incision to achieve a small surgical corridor. Following the initial dilatation, a series of dilators were consecutively inserted to retract the soft tissue sufficiently to accommodate the selected retractor (22 mm diameter, METRx tubular retractor system, Medtronic, Minneapolis, MN).The retractor was then placed over the sequential dilators and then passed through the incision. A flexible arm was then attached to the retractor. All the sequential dilatators were then removed. After establishing the operative window, the operating microscope was positioned through the window. The tumor was carefully mobilized, and its capsule was longitudinally incised along its greater diameter (Figures 3C-3D). Intraoperatively, nerve stimulation with electromyography was conducted. The muscles simulated were the gastrocnemius and the anterior tibia. There was no pathological activity recorded. Hemostasis was obtained using bipolar coagulation. The tumor was enucleated and totally resected (Figure 3E).

A comparison of the conventional and minimally invasive surgical techniques is provided in Table 1.

**Table 1:** Comparison of conventional and minimally invasive techniques for sciatic nerve schwannomas.

	Conventional approach	Minimal invasive technique
Incision size	Large 5-10 cm	Small 1-2 cm
Operative time	1-3 hours	0.5-1 hour
Tissue disruption	Greater muscle and soft tissue dissection	Minimal tissue disruption
Visualization	Direct visualization possible	Indirect visualization with the microscope
Equipment required	Standard surgical equipment	Tubular retractor METRx system
Postoperative pain	Greater postoperative pain	Less pain
Extent of resection	Gross total resection	Gross total resection
Cosmetic outcome	noticeable scarring	Improved cosmetic results
Hospital stay (average number of days)	7 days	3-5 days

## Discussion

Schwannomas and neurofibromas comprise the most common peripheral nerve sheath tumors [1]. These tumors arise from non-neural cells with neuroectodermal origin located in the periphery of nerve fascicles. Extracranial schwannomas mainly affect the brachial plexus (36%-44%) and the upper extremities (17.6%-25.8%) [1,7]. The posterior tibial nerve in the tarsal sinus and the common sciatic nerve are the most common sites of involvement in the lower extremities [1,8]. Although the schwannomas in upper limbs usually emerge as visible painless swelling in anterior compartments, they commonly involve the posterior compartment of lower limbs and, therefore, are usually diagnosed when they are big enough to compress the parent nerves [9,10]. The sciatic nerve is affected, either localized or all along its course, in less than 1% of cases [8,11]. They typically manifest with dull, intermittent pain originating from the posterior aspect of the mid-thigh, which radiates to the distal distribution of the sciatic nerve [8,11]. Because it is similar in nature to common sciatica or tarsal tunnel syndrome, it often leads to a misdiagnosis [12]. However, suspicion of SSs should be raised when patients do not benefit from standard medical or surgical treatments, or when physical examination and lumbosacral MRI reveal no relevant findings. In approximately 65% of schwannomas, a palpable, percussion-sensitive mass with a positive Tinel’s sign can be detected [1]. Sensory or motor deficits are uncommon and may occur in SSs larger than 4 cm [13]. Pain aggravated by sitting, exercise, or palpation of the mass has been reported in a few cases of SS [8,13].

In ultrasonography, an SS appears as a hypoechoic mass eccentrically located on a nerve trunk. Ultrasonography may be superior to MRI in the diagnosis of small SSs [14]. However, in large SSs, it can fail to demonstrate the mass-nerve relationship in more than 50% of cases [15,16].

It may also help distinguish SS from NF by indicating whether the fibrillar structure of the affected nerve is preserved or disrupted [8]. MRI, however, remains the method of choice for diagnosis and preoperative surgical planning of soft-tissue neoplasms. In SS, MRI typically reveals a well-defined mass along the course of the sciatic nerve, showing an isointense signal on T1-weighted images and a hyperintense signal on T2-weighted images. Gadolinium administration results in intense, homogeneous enhancement in most cases [8]. SS in patients with NF1 has been very rarely reported to date [3,16]. Schwannomas most commonly occur sporadically, with only about 10% of cases arising in patients with NF2 [1]. In contrast, approximately 60% of patients with NF1 develop neurofibromas during their lifetime, which carry a 10%-15% risk of malignant transformation [17].

Herein, we report a patient with NF1 who developed early signs of SS within four months after resection of a lumbar schwannoma at the L1-L2 level. In this patient, the symptoms of SS may have been masked by the spinal tumor, as both presented with similar clinical features.

As SS rarely occurs in patients with NF1, peripheral nerve sheath tumors in this setting should be considered as NF2 until proven otherwise [17]. MRI features suggestive of SS include eccentric association with the nerve, a high-intensity peripheral rim, and preserved fascicular architecture [18]. Preoperative positron emission tomography (PET) has recently been recommended to exclude malignancy, although schwannomas may produce a similar pattern that is indistinguishable from malignant lesions [1]. Compared to SS, NFs more commonly involve the motor fascicles of mixed nerves, lack a peripheral capsule, and grow concentrically within fascicles, making radical resection with preservation of the sciatic nerve’s continuity and function difficult to achieve [1,7,8]. In a series of 182 benign peripheral nerve sheath tumors, the overall gross total resection (GTR) rates were 76.7% for schwannomas and 44.9% for neurofibromas [1]. In reported cases, these tumors have been resected via open exposure of the affected nerve through long longitudinal incisions, using different surgical approaches, such as posterior median, infragluteal, or transgluteal, depending on tumor size, location, and the surrounding neurovascular structures [19,20]. The large incision sizes and gluteal muscle traction used in these surgeries can result in large scars, muscle trauma, and collateral neurovascular injuries [20].

To the best of our knowledge, only one report of minimally invasive resection of SS exists in the literature [21]. A comparison between the conventional and minimally invasive techniques is presented in Table 1.

Taking this into account, a minimally invasive tubular approach was used to resect the tumor. Using sequential dilatators, the gluteal muscle was retracted, and finally, the tumor was exposed. The tumor was well-circumscribed, encapsulated, and eccentrically located on the sciatic nerve. The tumor was completely resected under neurophysiologic monitoring (Figure 3E). Histopathologic examination revealed the typical features of a schwannoma, including the hypercellular fibrillary Antoni A regions and the hypocellular Antoni B regions. The postoperative course was uneventful, with no neurologic deficits. At the most recent follow-up, eight years after surgery, there were no signs or symptoms of tumor recurrence, and the sensorimotor function of the affected sciatic nerve remained intact.

## Conclusions

Patients with NF1 classically present with two or more characteristic features, including café-au-lait macules, axillary or inguinal freckling, Lisch nodules, peripheral neurofibromas, and optic gliomas. Schwannomas occurring in patients with NF1 are rare, as NF1 is typically associated with neurofibromas rather than schwannomas. In this case report, the patient with NF1, in addition to the classical clinical stigmata, presented with schwannomas involving both the spine and the sciatic nerve. A minimally invasive tubular approach was used to resect the SS to reduce postoperative pain and shorten recovery time. Further studies with larger case numbers are necessary to establish the superiority of minimally invasive techniques over conventional surgical approaches. This case underscores the importance of considering schwannomas in the differential diagnosis of sciatica, even in patients with NF1, despite their rarity in this population. Prompt recognition and appropriate imaging facilitate timely surgical management and optimal outcomes.
